# Serum interleukin-6 level is correlated with the disease activity of systemic lupus erythematosus: a meta-analysis

**DOI:** 10.6061/clinics/2020/e1801

**Published:** 2020-10-05

**Authors:** Jianwen Ding, Shujun Su, Tao You, Tingting Xia, Xiaoying Lin, Zhaocong Chen, Liqun Zhang

**Affiliations:** I Department of Kidney Disease, Lanzhou University Second Hospital, Lanzhou 730030, China; II Department of Gynecology, The Third Affiliated Hospital, Sun Yat-sen University, Guangzhou, China; III Department of Endocrinology and Rheumatology, Affiliated Southeast Hospital of Xiamen University/909th Hospital of People's Liberation Army, 269 Zhanghua Middle Road, Zhangzhou, 363000, Fujian Province, China; IV Center for Reproductive Medicine, The Third Affiliated Hospital of Sun Yat-sen University, Guangzhou city, Guangdong Province, China; V Department of Rehabilitation Medicine, The Third Affiliated Hospital of Sun Yat-sen University, Guangzhou, Guangdong 510630, China

**Keywords:** Interleukin-6, Systemic Lupus Erythematosus, Autoimmune Disease

## Abstract

Interleukin-6 (IL-6) plays a crucial role in systemic autoimmunity and pathologic inflammation. Numerous studies have explored serum IL-6 levels in systemic lupus erythematosus (SLE) and their correlation with disease activity. Here, we performed a meta-analysis to quantitatively assess the correlation between the serum IL-6 levels and SLE activity.

The PubMed and EMBASE databases were thoroughly searched for relevant studies up to September 2019. Standardized mean differences (SMDs) with 95% confidence intervals (95% CIs) were used to describe the differences between serum IL-6 levels in SLE patients and healthy controls and between those in active SLE patients and inactive SLE patients. The correlation between the serum IL-6 levels and disease activity was evaluated using Fisher’s z values.

A total of 24 studies involving 1817 SLE patients and 874 healthy controls were included in this meta-analysis. Serum IL-6 levels were significantly higher in SLE patients than in the healthy controls (pooled SMD: 2.12, 95% CI: 1.21-3.03, Active SLE patients had higher serum IL-6 levels than inactive SLE patients (pooled SMD: 2.12, 95% CI: 1.21-3.03). Furthermore, the pooled Fisher’s z values (pooled Fisher’s z=0.36, 95% CI: 0.26-0.46, *p*<0.01) showed that there was a positive correlation between the serum IL-6 levels and SLE activity.

This study suggested that serum IL-6 levels were higher in patients with SLE than in healthy controls, and they were positively correlated with disease activity when Systemic Lupus Erythematosus Disease Activity Index>4 was defined as active SLE. More homogeneous studies with large sample sizes are warranted to confirm our findings due to several limitations in our meta-analysis.

## INTRODUCTION

Systemic lupus erythematosus (SLE) is a common inflammatory autoimmune disease that mainly occurs in reproductive age women (1). SLE can impair all organ systems, resulting in serious morbidities (2). Although its etiology remains elusive, it has been widely accepted that autoantibody production, immune complex deposition, and complement activation play key roles in SLE pathogenesis. Additionally, excessive pro-inflammatory cytokine release may also promote SLE development (3-6).

Interleukin-6 (IL-6) is a multifunctional pro-inflammatory cytokine, which can be secreted from endothelial cells, macrophages, lymphocytes, dendritic cells and fibroblasts (7). IL-6 can directly induce naïve B cell maturation into plasma cells (8) and facilitate cytotoxic T cell differentiation by upregulating IL-2 and IL-2R (8). Due to these functions, IL-6 promotes systemic autoimmunity and pathologic inflammatory responses (9,10). Numerous studies have reported that patients with SLE had higher serum IL-6 levels than healthy controls (11-13), suggesting that IL-6 might be involved in SLE pathogenesis. Moreover, evidence showed that higher serum IL-6 expression was significantly associated with SLE activity. Nevertheless, it has also been suggested that serum IL-6 levels might not be correlated with SLE disease activity. Therefore, we conducted this meta-analysis to ascertain the correlation between serum IL-6 levels and SLE activity.

## METHODS

### Search strategy

A systematic search was conducted for potential literature in two databases: PubMed and EMBASE (up to September 2019) . The key words used for the searches were as follows: “Interleukin-6 OR IL-6 OR Interleukin 6 OR IL6” and “systemic lupus erythematosus OR SLE.” No language or region limitations were used in the literature search.

### Study selection

Articles were included based on the following criteria: 1) case-control, cohort, or cross-sectional study designs in humans; 2) reports regarding details of serum IL-6 levels in SLE patients compared to those in normal controls and/or in SLE patients with active disease compared to inactive disease; 3) no limitations with regard to the definition of SLE activity; 4) reports regarding the mean and standard deviation/error of serum IL-6 levels.

The exclusion criteria included: 1) abstracts, case reports, animal studies, and reviews; 2) no full-text studies; 3) overlapping data; 4) insufficient data for meta-analysis, defined as reporting serum IL-6 levels without the mean and SD/SE.

### Data extraction and quality assessment

The following data were extracted independently by two investigators: first author, publication year, country, sample size of active/inactive SLE patients and the matched normal controls, mean age, percent of females in the sample population, mean and the standard deviation of IL-6 levels of two groups (pg/mL), detection method, definition of active SLE, and Pearson/Spearman correlation coefficient between the IL-6 level and SLE disease activity.

The quality of included studies was assessed using the Newcastle-Ottawa scale (NOS), and the high-quality articles were defined as those with a score higher than 5.

### Statistical analysis

The relationship between serum IL-6 levels and SLE was evaluated in the meta-analysis. For the continuous outcomes, we used standardized mean differences (SMDs) with 95% confidence intervals (95% CIs). For correlation coefficients, the published Spearman correlation coefficient (r) values were converted to Pearson correlation coefficient (r) values, which were used for the meta-analysis (14). After a Fisher *r-to-z* transformation for Pearson correlation coefficients (15,16), the pooled correlation coefficient results were presented as Fisher’s z values with the 95% CIs (17). *p*<0.05 was considered statistically significant. The Q-statistic and I^2^ statistic test were used to evaluate heterogeneity. Significant heterogeneity was defined based on the criteria: P_Q_<0.1 or I^2^>50%, and a random-effects model was used to calculate the pooled results. Otherwise, a fixed-effects model was applied. To detect the increased heterogeneity, subgroup analysis was performed according to the region, age, and the IL-6 assay method. Sensitivity analysis was conducted to evaluate the influence of each study on the pooled results. Publication bias was assessed using funnel plots and the Begg’s and Egger’s tests (18-21). The symmetric of funnel plots or the *p*<0.05 of the Begg’s or Egger’s tests indicated statistically significant publication bias, and the ‘‘trim-and-fill’’ method was used to adjust the summary estimates (22). All heterogeneity, meta-analysis, and sensitivity tests of the extracted data were performed using Review Manager 5.3 (Review Manager, 2014). Publication bias assessment and ‘‘trim-and-fill’’ analysis was conducted using STATA version 12.0 (Stata Corporation, College Station, TX, United States).

## RESULTS

### Literature selection

**Figure 1.**
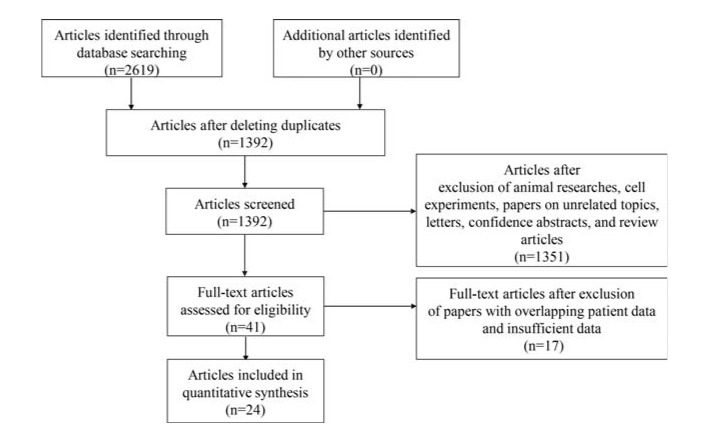
Flow chart of the selection process for eligible studies.

A total of 2619 articles were initially collected from PubMed and EMBASE. First, we excluded 1227 duplicate publications using EndNote X7 software. In next step, we reviewed titles and abstracts to further exclude 1351 articles featuring animal studies, cell experiments, unrelated topics, letters, and conference abstracts, and review articles. Then, we carefully reviewed the full text of the remaining 41 studies, removing 17 studies due to overlapping patient data and insufficient data. Finally, a total of 24 eligible studies were included in this meta-analysis (23-46). The flow chart of literature selection is presented in Figure 1.

### Characteristics of eligible studies

A total of 24 eligible studies with 1817 SLE patients and 874 healthy controls were included in this meta-analysis (23-46). These studies were published from 1996 to 2019. There were 12 studies enrolling patients from Asian countries (China (28,29,43,46), Japan (42), South Korea (26), Iran (36), Singapore (34), Malaysia (45), Thailand (44), and Turkey (32,33)). The serum IL-6 levels in most eligible studies were measured using enzyme-linked immunosorbent assay (ELISA) kits (23,24,26-29,31-33,37-39,41,43-45). The serum IL-6 levels ranged from 4.272±0.4222 to 123.71±81.783 (pg/mL) in SLE patients and 0.93±0.95 to 10.46±4.33 (pg/mL) in healthy controls. A total of 13 studies compared the serum IL-6 levels between SLE patients and healthy controls (23-25,27-29,32-34,39,40,43,46). Serum IL-6 levels between active and inactive SLE patients were compared in 9 studies (24-26,29,31,39,41-43). Additionally, the correlation coefficient between serum IL-6 level and SLE disease activity was reported in 13 studies (24-26,29-31,35-39,44,45). Among all included studies, 3 studies focused on children with SLE (25,28,46), and 21 studies focused on adults with SLE (23,24,26,27,29-45). The NOS scores for the included studies ranged from six to seven, indicating that they were of moderate to high quality and suitable for pooled analysis. The characteristics of the eligible studies are summarized in Table 1.

### Meta-analysis comparing the serum IL-6 levels in SLE patients and healthy controls

**Figure 2.**
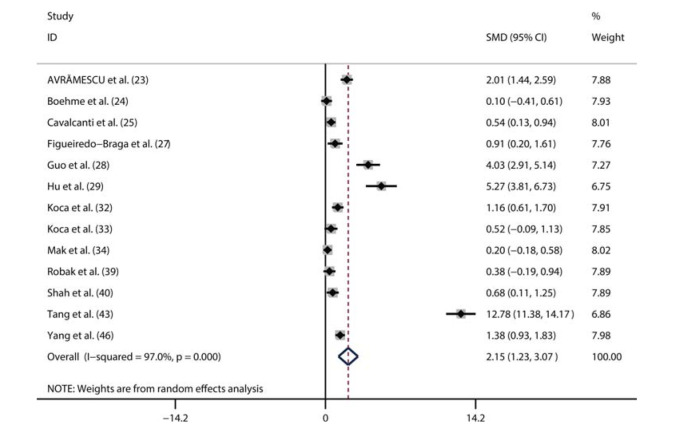
Meta-analysis comparing the serum IL-6 levels in SLE patients and healthy controls.

A total of 13 studies compared serum IL-6 levels between SLE patients (n=612) and healthy controls (n=376) (23-25,27-29,32-34,39,40,43,46). Among the 612 patients, 465 were female and 148 were male. Additionally, seven studies were from Asia, and six studies were conducted outside Asia. Moreover, ten studies only included adult patients with SLE, while three studies enrolled 161 children with SLE. Considering the significant heterogeneity (I^2^=97%, *p*<0.01), we used a random-effects model to perform the pooled analysis. We found that the serum IL-6 level was markedly higher in SLE patients than in healthy controls (pooled SMD: 2.12, 95% CI: 1.21-3.03, *p*<0.01) (Figure 2).

### Meta-analysis of the correlation between the serum IL-6 level and SLE activity

**Figure 3.**
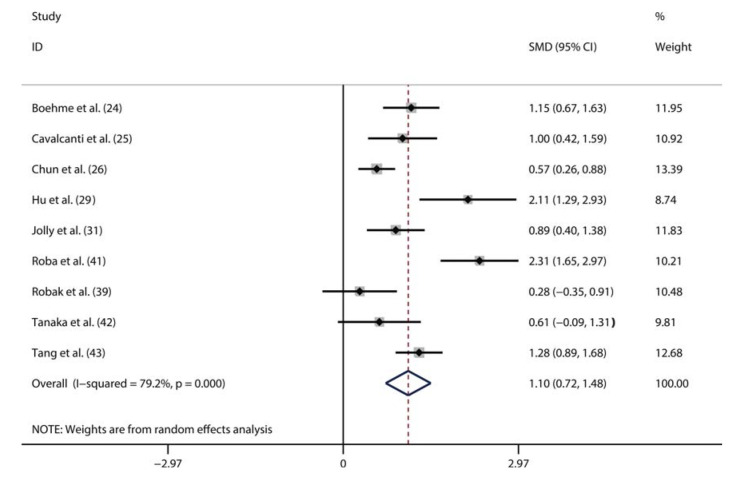
Meta-analysis comparing the serum IL-6 levels in active SLE patients and inactive SLE patients.

**Figure 4.**
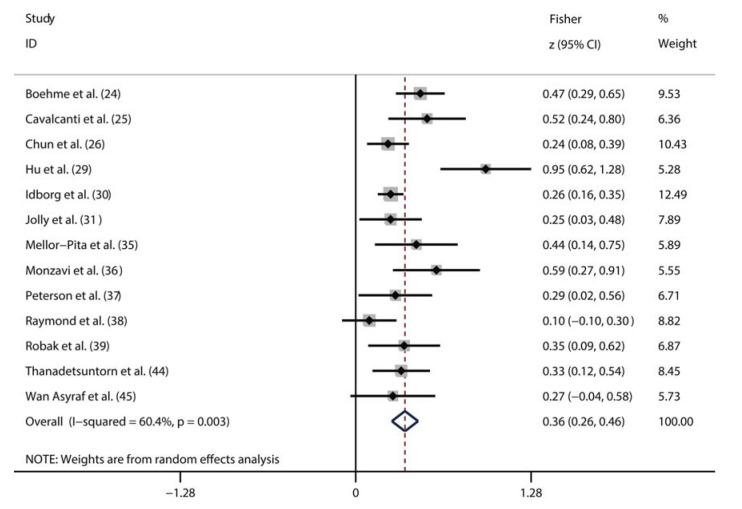
Meta-analysis of the correlation between the serum IL-6 level and SLE activity.

A total of nine studies compared the serum IL-6 levels between patients with active SLE and inactive SLE (24-26,29,31,39,41-43). The random-effects model was used to perform the pooled analysis due to the significant heterogeneity (I^2^=96%, *p*<0.01). We found that the serum IL-6 level was significantly higher in active SLE patients than in the inactive SLE patients (pooled SMD: 2.12, 95% CI: 1.21-3.03) (Figure 3). Additionally, 13 studies assessed the correlation between the serum IL-6 level and SLE activity using correlation coefficients (24-26,29-31,35-39,44,45). Considering the significant heterogeneity (I^2^=60%, *p*<0.01), we also pooled these data using a random-effects model. As shown in Figure 4, there was a positive correlation between the serum IL-6 level and SLE activity (Fisher’s z=0.36, 95% CI: 0.26-0.46, *p*<0.01).

### Subgroup analysis

To explore the source of heterogeneity for the overall pooled results, we conducted subgroup analyses based on age, region, assay method, and definition of disease activity. The results showed that SLE patients had a higher serum IL-6 level than healthy controls (Table 2), and the serum IL-6 level was positively correlated with SLE activity (Table 3) in the subgroup analyses with regard to the age, region, and assay method. Meanwhile, we observed that significant heterogeneity still existed in these subgroup analyses (Table 2 and 3). Thus, age, region, or assay method might not be a source of heterogeneity. Notably, subgroup analysis based on the definition of disease activity yielded inconsistent results. When Systemic Lupus Erythematosus Disease Activity Index 2000 (SLEDAI-2K)>4 was defined as active disease, no correlation between serum IL-6 level and SLE activity was identified (Table 3). In contrast, the correlation of serum IL-6 level with SLE activity was significant when active disease was defined as Systemic Lupus Erythematosus Disease Activity Index (SLEDAI)>4 (Table 3). Moreover, we found that there was no statistically significant heterogeneity in the subgroup with SLEDAI>4 (Table 3). These results indicated that the definition of disease activity might be a source of heterogeneity for the correlation between serum IL-6 level and SLE activity.

### Sensitivity analysis and publication bias

**Figure 5.**
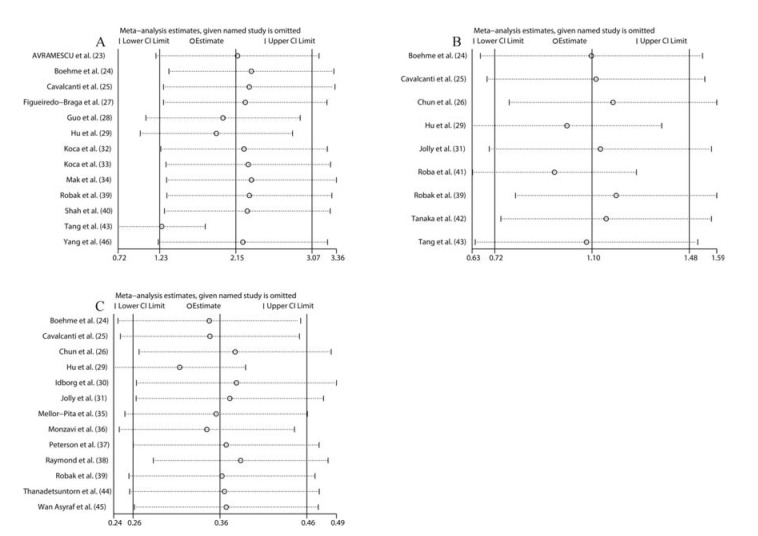
Sensitivity analysis for the pooled results of differences between the serum IL-6 levels in SLE patients and healthy controls (A); Sensitivity analysis for the pooled results of the differences between the serum IL-6 level in active SLE patients and inactive SLE patients (B); Sensitivity analysis for the pooled results of the correlation between the serum IL-6 level and SLE activity (C).

**Figure 6.**
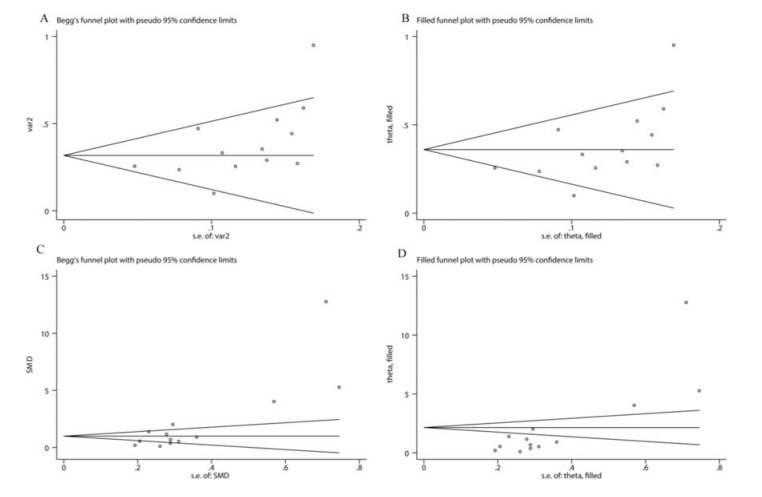
Funnel plot of the pooled results of differences between serum IL-6 levels in SLE patients and healthy controls (A); The adjusted funnel plot of the pooled results of differences between serum IL-6 levels in SLE patients and healthy controls (B); Funnel plot of the pooled results of the correlation between the serum IL-6 level and SLE activity (C); The adjusted funnel plot of the pooled results of the correlation between the serum IL-6 level and SLE activity (D).

Sensitivity analysis was performed by sequentially deleting a single study to explore the source of heterogeneity. As shown in Figure 5, the overall pooled SMDs were not significantly altered. Egger’s tests and Begg’s tests were conducted to evaluate publication bias. As illustrated in Figure 6A, the funnel plot from Begg’s tests was asymmetric. Moreover, the *p* values of Egger’s tests *(p=*0.001) and Begg’s tests *(p=*0.005) were less than 0.05. These results suggested that there was significant publication bias. Subsequently, we utilized the trim-and-fill method to examine whether publication bias substantially affected the robustness of the pooled SMD. The results showed that the adjusted funnel plot became symmetric (Figure 6B). Meanwhile, the pooled SMD (SMD: 2.15, 95% CI: 1.23-3.07) obtained after trim-and-fill adjustment still showed that the serum IL-6 level was higher in SLE patients than in healthy controls, suggesting that publication bias did not substantially affect the robustness of the pooled SMD. For the pooled Fisher’s z value assessing the correlation between serum IL-6 level and SLE activity, Egger’s tests *(p=*0.056) showed no significant publication bias, but Begg’s tests *(p=*0.02) indicated that significant publication bias existed. Additionally, the funnel plot in Begg’s test was also asymmetric (Figure 6C), further confirming that there was significant publication bias. Next, we applied the trim-and-fill method to determine whether the robustness of the pooled Fisher’s z value was influenced by publication bias. As shown in Figure 6D, the adjusted funnel plot became symmetric, indicating that publication bias did not significantly affect the robustness of the pooled Fisher’s z value. Consistently, the pooled Fisher’s z value (0.360, 95% CI: 0.264-0.456, *p*<0.01) after trim-and-fill adjustment still suggested that there was a positive correlation between serum IL-6 level and SLE activity.

## DISCUSSION

To the best of our knowledge, this study is the first meta-analysis to quantitatively evaluate the correlation between the serum IL-6 level and SLE. In this meta-analysis, we found that the serum IL-6 level in SLE patients was significantly higher than that in healthy controls. Additionally, our overall pooled results showed that the serum IL-6 level was positively correlated with active SLE. However, our subgroup analysis found no correlation between serum IL-6 level and SLE activity when SLEDAI-2K>4 was defined as active disease. In contrast, the serum IL-6 level was positively correlated with SLE disease activity when active SLE was defined as SLEDAI>4.

Several mechanisms may explain the positive correlation between the serum IL-6 levels and SLE. Autoantibody overproduction can cause severe immune complex deposition, which largely promotes SLE pathogenesis. It has been suggested that IL-6 may increase autoantibody production by promoting autoreactive B lymphocyte proliferation (47) and the differentiation of naive B cells into plasma cells (48). Additionally, IL-6 could upregulate the expression of recombination-activating genes and promote V(D)J rearrangement, which also leads to autoantibody overproduction in SLE (49). Evidence has revealed that Th17/Treg ratio imbalance is involved in the development of various autoimmune diseases. Interestingly, it has been found that IL-6 could promote the differentiation of naïve CD4+ T cells into Th17 cells by activating the STAT3 pathway, but IL-6 could impair Treg differentiation (50), suggesting that IL-6 may also contribute to SLE development by meditating the Th17/Treg imbalance. Notably, a previous study revealed that IL-6 could increase vascular permeability by inducing the secretion of vascular endothelial growth factor from fibroblast-like synoviocytes (51), promoting inflammatory cell infiltration and immune complex deposition. Thus, IL-6 may also promote SLE development by impairing the vascular endothelial function. Taken together, this evidence supports our findings that high serum IL-6 levels are positively correlated with SLE disease activity.

Our findings in this study may have clinical significance for SLE treatment. First, physicians may monitor SLE disease activity using the serum IL-6 level. Additionally, our findings revealed that IL-6-targeted therapy could be an effective strategy for SLE therapy. Notably, it has been demonstrated that using blocking antibodies against IL-6 and IL-6R could prevent SLE onset and progression in a mouse model of SLE (52,53). It has been suggested that T lymphocytes, monocytes, dendritic cells, and macrophages play key roles in promoting SLE development likely via the overproduction of IL-6 (35,54,55). Lu et al. revealed that HMGB1 could activate macrophages to secrete IL-6, facilitating the progression of SLE (54); this suggests that HMGB1 might be a risk factor for SLE. Moreover, manipulation of the HMGB1 signaling pathway may represent a novel SLE treatment strategy. However, few studies have been conducted to explore which factors promote IL-6 release by T lymphocytes, monocytes, and dendritic cells. Therefore, subsequent experimental studies are warranted to elucidate the mechanisms underlying IL-6 overproduction by T lymphocytes, monocytes, and dendritic cells to develop new therapeutic strategies for SLE.

There are several limitations in this meta-analysis that should be considered. First, most of the studies are case-control studies, in which bias may be unavoidable. In addition, significant publication bias also existed in our meta-analysis. Second, the pooled analysis showed significant heterogeneity. Based on subgroup analyses, the difference in the definition of disease activity might be a source of heterogeneity. More clinical studies with the identical definition of SLE activity should be conducted to further determine the correlation between serum IL-6 levels and SLE. Third, SLE is a heterogeneous disease causing damage to multiple organ systems. Therefore, it will be more clinically helpful to evaluate the correlation between IL-6 and specific manifestations such as nephritis and central nervous system involvement. Although a few studies suggested that serum IL-6 might be associated with nephritis (56) and central nervous system involvement (57), it is hard to perform the relevant pooled analyses due to the lack of sufficient data in the included studies. Fourth, previous studies showed that corticosteroid therapy could effectively inhibit IL-6 production in SLE (58,59), suggesting that the difference in immune-suppressor therapy may overestimate or underestimate the correlation between IL-6 and SLE. In this meta-analysis, most of the included studies did not report whether the SLE patients received corticosteroid therapy or therapy with other immune-suppressors before the sampling of blood to detect IL-6; hence, we could not obtain sufficient data to conduct the subgroup analysis to determine the reliability of the pooled analyses on the correlation between IL-6 and SLE. Fifth, although most of the included studies used ELISA to assess the IL-6 levels, some studies adopted a different methodology. In addition, the kits for ELISA analysis in different studies might be not identical. Evidently, these differences may result in heterogeneity among the studies. Sixth, the Fisher’s z values for correlating the IL-6 levels and SLE activity were transformed from the correlation coefficients, due to which minor calculation errors might be unavoidable; this probably affected the reliability of our pooled results. Seventh, the sample sizes were small in some subgroup analyses; thus, the reliability of the pooled results might be discounted.

In conclusion, our study suggested that serum IL-6 levels were higher in patients with SLE than in healthy controls. Furthermore, serum IL-6 levels were positively correlated with SLE disease activity when active SLE was defined using the criterion: SLEDAI>4. Therefore, the serum IL-6 level may be a promising biomarker to monitor SLE disease activity. More importantly, IL-6-targeted therapy could serve as an effective strategy for treating SLE patients. However, more homogeneous studies with large sample sizes are required to validate our findings, owing to several limitations in our meta-analysis.

## AUTHOR CONTRIBUTIONS

Zhang L, Chen Z and Ding J designed the study. You T, Su S, Xia T and Lin X performed data extraction and analysis and wrote the manuscript. Su S and Ding J were mainly responsible for revising this manuscript. All of the authors have read and approved the final version of the manuscript.

## Figures and Tables

**Table 1.** Characteristics of eligible studies.

Study	Country	SLE			Control			Detection methods	Definition of active SLE	NOS
		Sample size	mean age (y)	female (%)	Sample size	Mean age (y)	Female (%)			
		Active/Inactive	Active/Inactive	Active/Inactive						
AVRĂMESCU et al. (23)	Romania	35	>18	91.4	35	Matched	Matched	ELISA	NA	5
Boehme et al. (24)	Germany	30/56	34±6	86.7	20	34±9	70	ELISA	SLAM≥11	6
Cavalcanti et al. (25)	Brazil	26/25	15	92	47	15	91	CBA	SLEDAI-2K≥4	7
Chun et al. (26)	Korea	90/77	34.3±0.85	87.3	40	35.5±1.02	87.5	ELISA	SLEDAI≥6	7
Figueiredo-Braga et al. (27)	Portugal	15	49±8.2	100	20	43.95±11.77	95	ELISA	NA	6
Guo et al. (28)	China	22	13.6	95	17	14.2	94	ELISA	NA	6
Hu et al. (29)	China	24/14	30.5±8.6	89.5	20	33.6±7.8	75	ELISA	NA	6
Idborg et al. (30)	Sweden	322/115	47.2	92	322	48.2	92	NA	SLEDAI-2K>0	7
Jolly et al. (31)	USA	27/50	44.9±12.7	93.5	NA	NA	NA	ELISA	SLEDAI≥4	6
Koca et al. (32)	Turkey	28	33.4±8.8	96.4	33	41.9±13.7	54.5	ELISA	NA	5
Koca et al. (33)	Turkey	20	35.8±10.9	100	23	39.5±9.2	100	ELISA	SLEDAI≥6	5
Mak et al. (34)	Singapore	54	40.59±14.8	89	54	40.02±13.7	82	Millipore	NA	6
Mellor-pita et al. (35)	Spain	45	39.6±11.1	84.4	19	35±9.9	84.2	ELISA	SLEDAI>4	6
Monzavi et al. (36)	Iran	41	31	100	41	Matched	100	ELISA	NA	6
Peterson et al. (37)	USA	56	38	93	32	35	91	ELISA	NA	5
Raymond et al. (38)	Australia	58/42	49	87	31	50	77	ELISA	SLEDAI-2K>4	7
Roba 2015	Egypt	32/28	28.58±7.30	93.3	NA	NA	NA	ELISA	SLEDAI≥6	6
Robak et al. (39)	Poland	52/12	38	94	15	39	93	ELISA	SLAM>10	6
Shah et al. (40)	USA	25	37.7±11.4	100	26	Matched	Matched	BBP	NA	6
Tanaka et al. (42)	Japan	22/13	41.1±12.1/43.2±13.6	90.9/92.3	13	NA	NA	CBA	SLEDAI≥6	6
Tang et al. (43)	China	100/40	34.15±9.84/36.75±11.42	81/80	36	37.83±6.84	86.1	ELISA	SLEDAI-2K>4	7
Thanadetsuntorn et al. (44)	Thailand	27/63	32.67±13.34/43.35±14.24	96.8/85.2	NA	NA	NA	ELISA	Modified SLEDAI-2K>1	7
Wan Asyraf et al. (45)	Malaysia	12/31	30.5/34	97.7	NA	NA	NA	ELISA	SLEDAI≥4	5
Yang et al. (46)	China	23/65	10.03±2.11	88.6	30	9.81±2.23	70	ITA	SLEDAI>9	6

Abberviations: CBA: Cytometric Bead Array; Millipore: Miliplex Human Cytokine/Chemokine panel; BBP: Bio-Plex Pro human cytokine assay kit; ITA: Immunoturbidimetry; SLAM: the Systemic Lupus Activity Measure; SLEDAI: Systemic Lupus Erythematosus Disease Activity Index; SLEDAI-2K: Systemic Lupus Erythematosus Disease Activity Index 2000; NA: not available; NOS: Newcastle-Ottawa scale.

**Table 2.** Subgroup analyses for the pooled results of differences in serum IL-6 levels between SLE patients and healthy controls.

Variables	No. of studies	Pooled SMD (95% CI)	*p*-value	Heterogeneity	
				I^2^ (%)	*p*-value
**[1] Age**					
Adult	10	2.257 (1.054-3.46)	<0.01	97.5	<0.01
Child	3	1.861 (0.499-3.224)	<0.01	94.4	<0.01
**[2] Region**					
Non-Asia	6	0.757 (0.24-1.275)	<0.01	81.7	<0.01
Asia	7	3.508 (1.662-5.354)	<0.01	98.3	<0.01
**[3] Measuring methods**					
ELISA	9	2.914 (1.37-4.458)	<0.01	88.6	<0.01
Other methods	4	0.691 (0.181-1.201)	<0.01	37.5	0.2

**Table 3.** Subgroup and meta-regression analyses of the pooled results of the correlation between serum IL-6 levels and SLE disease severity.

Variables	No. of studies	Fisher’s z (95% CI)	*p*-value	Heterogeneity	
				I^2^ (%)	*p*-value
**[1] Age**					
Adult	12	0.349 (0.25-0.448)	<0.01	61	<0.01
Child	1	0.522 (0.239-0.805)	<0.01	-	-
**[2] Region**					
Non-Asia	8	0.317 (0.221-0.412)	<0.01	41.1	0.1
Asia	5	0.452 (0.221-0.682)	<0.01	76.6	<0.01
**[3] Measuring methods**					
ELISA	11	0.367 (0.251-0.483)	<0.01	61.9	<0.01
Other methods	2	0.354 (0.102-0.606)	<0.01	67.4	0.08
**[4] Definition of activity**					
SLEDAI-2K>4	2	0.298 (−0.115-0.711)	0.16	82.6	0.02
SLEDAI>4	3	0.31 (0.153-0.467)	<0.01	0	0.6

## References

[1] Johnson AE, Gordon C, Palmer RG, Bacon PA (1995). The prevalence and incidence of systemic lupus erythematosus in Birmingham, England. Relationship to ethnicity and country of birth. Arthritis Rheum.

[2] Ruiz-Irastorza G, Khamashta MA, Castellino G, Hughes GR (2001). Systemic lupus erythematosus. Lancet.

[3] Dean GS, Tyrrell-Price J, Crawley E, Isenberg DA (2000). Cytokines and systemic lupus erythematosus. Ann Rheum Dis.

[4] Aringer M, Smolen JS (2008). The role of tumor necrosis factor-alpha in systemic lupus erythematosus. Arthritis Res Ther.

[5] Tackey E, Lipsky PE, Illei GG (2004). Rationale for interleukin-6 blockade in systemic lupus erythematosus. Lupus.

[6] Pacheco Y, Barahona-Correa J, Monsalve DM, Acosta-Ampudia Y, Rojas M, Rodríguez Y (2017). Cytokine and autoantibody clusters interaction in systemic lupus erythematosus. J Transl Med.

[7] Lotz M (1995). Interleukin-6: a comprehensive review. Cancer Treat Res.

[8] Naka T, Nishimoto N, Kishimoto T (2002). The paradigm of IL-6: from basic science to medicine. Arthritis Res.

[9] Kimura A, Kishimoto T (2010). IL-6: regulator of Treg/Th17 balance. Eur J Immunol.

[10] Yuk CM, Park HJ, Kwon BI, Lah SJ, Chang J, Kim JY (2017). Basophil-derived IL-6 regulates TH17 cell differentiation and CD4 T cell immunity. Sci Rep.

[11] Fraunberger P, Wang Y, Holler E, Parhofer KG, Nagel D, Walli AK (2006). Prognostic value of interleukin 6, procalcitonin, and C-reactive protein levels in intensive care unit patients during first increase of fever. Shock.

[12] Mroczko B, Groblewska M, Gryko M, Kedra B, Szmitkowski M (2010). Diagnostic usefulness of serum interleukin 6 (IL-6) and C-reactive protein (CRP) in the differentiation between pancreatic cancer and chronic pancreatitis. J Clin Lab Anal.

[13] Panichi V, Maggiore U, Taccola D, Migliori M, Rizza GM, Consani C (2004). Interleukin-6 is a stronger predictor of total and cardiovascular mortality than C-reactive protein in haemodialysis patients. Nephrol Dial Transplant.

[14] Rupinski MT, Dunlap WP (1996). Approximating Pearson product-moment correlations from Kendall's tau and Spearman's rho. Educational and Psychological Measurement.

[15] Fisher RA (1921). On the “probable error” of a coefficient of correlation deduced from a small sample. Metron.

[16] Fisher RA (1915). Frequency Distribution of the Values of the Correlation Coefficient in Samples from an Indefinitely Large Population. Biometrika.

[17] Shadish WR, Haddock CK, Cooper H., Hedges L. V. (1994). Combining estimates of effect size.. The handbook of research synthesis.

[18] Egger M, Davey Smith G, Schneider M, Minder C (1997). Bias in meta-analysis detected by a simple, graphical test. BMJ.

[19] Begg CB, Mazumdar M (1994). Operating characteristics of a rank correlation test for publication bias. Biometrics.

[20] Xiao Y, Liu H, Chen L, Wang Y, Yao X, Jiang X (2019). Association of microRNAs genes polymorphisms with arthritis: a systematic review and meta-analysis. Biosci Rep.

[21] Ye J, Sun H, Feng Z, Zhang Q, Xia Y, Ji Y (2019). Prognostic significance of LncRNA GHET1 expression in various cancers: a systematic review and meta-analysis. Biosci Rep.

[22] Duval S, Tweedie R (2000). Trim and fill: A simple funnel-plot-based method of testing and adjusting for publication bias in meta-analysis. Biometrics.

[23] Avrămescu C, Biciuşcă V, Dăianu T, Turculeanu A, Bălăşoiu M, Popescu SN (2010). Cytokine panel and histopathological aspects in the systemic lupus erythematosus. Rom J Morphol Embryol.

[24] Boehme MW, Raeth U, Galle PR, Stremmel W, Scherbaum WA (2000). Serum thrombomodulin-a reliable marker of disease activity in systemic lupus erythematosus (SLE): advantage over established serological parameters to indicate disease activity. Clin Exp Immunol.

[25] Cavalcanti A, Santos R, Mesquita Z, Duarte AL, Lucena-Silva N (2017). Cytokine profile in childhood-onset systemic lupus erythematosus: a cross-sectional and longitudinal study. Braz J Med Biol Res.

[26] Chun HY, Chung JW, Kim HA, Yun JM, Jeon JY, Ye YM (2007). Cytokine IL-6 and IL-10 as biomarkers in systemic lupus erythematosus. J Clin Immunol.

[27] Figueiredo-Braga M, Cornaby C, Cortez A, Bernardes M, Terroso G, Figueiredo M (2018). Depression and anxiety in systemic lupus erythematosus: The crosstalk between immunological, clinical, and psychosocial factors. Medicine.

[28] Guo Y, Chai Q, Zhao Y, Li P, Qiao J, Huang J (2015). Increased activation of toll-like receptors-7 and -8 of peripheral blood mononuclear cells and upregulated serum cytokines in patients with pediatric systemic lupus erythematosus. Int J Clin Exp Med.

[29] Hu S, Xu Q, Xiao W, Huang M (2006). The expression of molecular chaperone HSP90 and IL-6 in patients with systemic lupus erythematosus. J Huazhong Univ Sci Technolog Med Sci.

[30] Idborg H, Eketjäll S, Pettersson S, Gustafsson JT, Zickert A, Kvarnström M (2018). TNF-α and plasma albumin as biomarkers of disease activity in systemic lupus erythematosus. Lupus Sci Med.

[31] Jolly M, Francis S, Aggarwal R, Mikolaitis RA, Niewold TB, Chubinskaya S (2014). Serum free light chains, interferon-alpha, and interleukins in systemic lupus erythematosus. Lupus.

[32] Koca SS, Isik A, Ustundag B, Metin K, Aksoy K (2008). Serum pro-hepcidin levels in rheumatoid arthritis and systemic lupus erythematosus. Inflammation.

[33] Koca SS, Özgen M, Işık B, Dağlı MN, Üstündağ B, Işık A (2014). Serum salusin-α levels in systemic lupus erythematosus and systemic sclerosis. Eur J Rheumatol.

[34] Mak A, Tang CS, Ho RC (2013). Serum tumour necrosis factor-alpha is associated with poor health-related quality of life and depressive symptoms in patients with systemic lupus erythematosus. Lupus.

[35] Mellor-Pita S, Citores MJ, Castejon R, Yebra-Bango M, Tutor-Ureta P, Rosado S (2009). Monocytes and T lymphocytes contribute to a predominance of interleukin 6 and interleukin 10 in systemic lupus erythematosus. Cytometry B Clin Cytom.

[36] Monzavi SM, Alirezaei A, Shariati-Sarabi Z, Tavakol Afshari J, Mahmoudi M, Dormanesh B (2018). Efficacy analysis of hydroxychloroquine therapy in systemic lupus erythematosus: a study on disease activity and immunological biomarkers. Inflammopharmacology.

[37] Peterson E, Robertson AD, Emlen W (1996). Serum and urinary interleukin-6 in systemic lupus erythematosus. Lupus.

[38] Raymond WD, Eilertsen GO, Nossent J (2019). Principal component analysis reveals disconnect between regulatory cytokines and disease activity in Systemic Lupus Erythematosus. Cytokine.

[39] Robak E, Sysa-Jedrzejowska A, Stepień H, Robak T (1997). Circulating interleukin-6 type cytokines in patients with systemic lupus erythematosus. Eur Cytokine Netw.

[40] Shah K, Lee WW, Lee SH, Kim SH, Kang SW, Craft J (2010). Dysregulated balance of Th17 and Th1 cells in systemic lupus erythematosus. Arthritis Res Ther.

[41] Talaat RM, Mohamed SF, Bassyouni IH, Raouf AA (2015). Th1/Th2/Th17/Treg cytokine imbalance in systemic lupus erythematosus (SLE) patients: Correlation with disease activity. Cytokine.

[42] Tanaka A, Ito T, Kibata K, Inagaki-Katashiba N, Amuro H, Nishizawa T (2019). Serum high-mobility group box 1 is correlated with interferon-α and may predict disease activity in patients with systemic lupus erythematosus. Lupus.

[43] Tang Y, Tao H, Gong Y, Chen F, Li C, Yang X (2019). Changes of Serum IL-6, IL-17, and Complements in Systemic Lupus Erythematosus Patients. J Interferon Cytokine Res.

[44] Thanadetsuntorn C, Ngamjanyaporn P, Setthaudom C, Hodge K, Saengpiya N, Pisitkun P (2018). The model of circulating immune complexes and interleukin-6 improves the prediction of disease activity in systemic lupus erythematosus. Sci Rep.

[45] Wan Asyraf WA, Mohd Shahrir MS, Asrul W, Norasyikin AW, Hanita O, Kong WY (2018). The association between serum prolactin levels and interleukin-6 and systemic lupus erythematosus activity. Reumatismo.

[46] Yang Y, Che Y, Yang L (2019). Relationship of serum inflammatory cytokines with anemia and vascular endothelial function in children with systemic lupus erythematosus. Clin Hemorheol Microcirc.

[47] Linker-Israeli M, Deans RJ, Wallace DJ, Prehn J, Ozeri-Chen T, Klinenberg JR (1991). Elevated levels of endogenous IL-6 in systemic lupus erythematosus. A putative role in pathogenesis. J Immunol.

[48] Kitani A, Hara M, Hirose T, Harigai M, Suzuki K, Kawakami M (1992). Autostimulatory effects of IL-6 on excessive B cell differentiation in patients with systemic lupus erythematosus: analysis of IL-6 production and IL-6R expression. Clin Exp Immunol.

[49] Hillion S, Garaud S, Devauchelle V, Bordron A, Berthou C, Youinou P (2007). Interleukin-6 is responsible for aberrant B-cell receptor-mediated regulation of RAG expression in systemic lupus erythematosus. Immunology.

[50] Acosta-Rodriguez EV, Napolitani G, Lanzavecchia A, Sallusto F (2007). Interleukins 1beta and 6 but not transforming growth factor-beta are essential for the differentiation of interleukin 17-producing human T helper cells. Nat Immunol.

[51] Nakahara H, Song J, Sugimoto M, Hagihara K, Kishimoto T, Yoshizaki K (2003). Anti-interleukin-6 receptor antibody therapy reduces vascular endothelial growth factor production in rheumatoid arthritis. Arthritis Rheum.

[52] Mihara M, Kasutani K, Okazaki M, Nakamura A, Kawai S, Sugimoto M (2005). Tocilizumab inhibits signal transduction mediated by both mIL-6R and sIL-6R, but not by the receptors of other members of IL-6 cytokine family. Int Immunopharmacol.

[53] Liang B, Gardner DB, Griswold DE, Bugelski PJ, Song XY (2006). Anti-interleukin-6 monoclonal antibody inhibits autoimmune responses in a murine model of systemic lupus erythematosus. Immunology.

[54] Lu M, Yu S, Xu W, Gao B, Xiong S (2015). HMGB1 Promotes Systemic Lupus Erythematosus by Enhancing Macrophage Inflammatory Response. J Immunol Res.

[55] Decker P, Kötter I, Klein R, Berner B, Rammensee HG (2006). Monocyte-derived dendritic cells over-express CD86 in patients with systemic lupus erythematosus. Rheumatology.

[56] El-Shereef RR, Lotfi A, Abdel-Naeam EA, Tawfik H (2016). Serum and Urinary Interleukin-6 in Assessment of Renal Activity in Egyptian Patients with Systemic Lupus Erythematosus. Clin Med Insights Arthritis Musculoskelet Disord.

[57] Fragoso-Loyo H, Richaud-Patin Y, Orozco-Narváez A, Dávila-Maldonado L, Atisha-Fregoso Y, Llorente L (2007). Interleukin-6 and chemokines in the neuropsychiatric manifestations of systemic lupus erythematosus. Arthritis Rheum.

[58] Cepika AM, Bendelja K, Vergles JM, Malenica B, Kapitanovic S, Gagro A (2010). Monocyte response to LPS after exposure to corticosteroids and chloroquine with implications for systemic lupus erythematosus. Scand J Immunol.

[59] Shemer A, Kivity S, Shovman O, Bashi T, Perry O, Watad A (2018). Tuftsin-phosphorylcholine (TPC) equally effective to methylprednisolone in ameliorating lupus nephritis in a mice model. Clin Exp Immunol.

